# Joint Angular Excursions and Angular Range Utilization During Stance‐Phase Locomotion in Terrestrial Mammals: A Comparative Morphofunctional Data Set

**DOI:** 10.1002/jez.70069

**Published:** 2026-02-09

**Authors:** Paul Medina‐González

**Affiliations:** ^1^ Departamento de Kinesiología, Facultad de Ciencias de la Salud Universidad Católica del Maule Talca Chile

**Keywords:** angular range utilization, biomechanics, functional morphology, joint angular excursion, limb posture, paleobiology, terrestrial mammals

## Abstract

Morphofunctional inferences based on anatomical structure often rely on static skeletal features, with limited integration of dynamic locomotor behavior. Although mammalian limb movement exhibits conserved kinematic synergies, to our knowledge no broad comparative data set has quantified how joint poses, angular excursions, and angular range utilization vary across biological factors. A comparative data set of joint motion during the stance phase of walking is presented for 182 terrestrial mammal species spanning 15 orders, classified by limb posture, body mass, top speed, and locomotor habit. Using sagittal‐plane video analysis and published sources, joint angles at touchdown, midstance, and toe‐off were measured for six major limb joints. From these data, joint angular excursion (JAE), total angular excursion (TAE), and an angular utilization index (AUI% = TAE/∑JAE) expressed as the percentage of summed joint excursion that is realized as net limb excursion during stance, were calculated. Using phylogenetic generalized least squares (PGLS) to account for nonindependence among species, I found that JAE and TAE covaried with the factors considered, with body mass emerging as the dominant predictor. Hindlimb and forelimb TAE decreased with increasing log_10_ body mass, whereas posture effects were subtle and largely overlapping among categories. Plantigrade, small‐bodied and arboreal species tended to display broader angular profiles, whereas unguligrade, cursorial and fast‐moving taxa generally used smaller excursions. Quadrant‐based comparisons of forelimb and hindlimb AUI further highlighted locomotor strategies aligned with biological factors. Together, these findings indicate that mammals modulate the magnitude and distribution of joint excursions across size and ecological gradients while broadly preserving the proportion of the summed joint excursions range used during stance, providing a reproducible framework for interpreting limb dynamics in extant and extinct mammals.

## Summary

- Joint and limb angular excursions during stance vary across biological factors, with body mass emerging as the dominant predictor.
- Despite differences in absolute excursion, angular utilization (AUI%) remains broadly conserved across mammals, consistent with shared neuromechanical coordination.
- This comparative kinematic framework provides a baseline for probabilistic morphofunctional inference, including locomotor evolution and fossil gait reconstruction.

## Graphical abstract

This study quantifies stance‐phase joint angular excursions and angular utilization (AUI%) during walking in 182 terrestrial mammal species. Across mammals, total limb excursion during stance (TAE) decreases with increasing body mass, whereas AUI% remains broadly conserved, indicating similar proportional use of summed joint excursions to generate net limb excursion. These patterns provide a comparative kinematic baseline to support probabilistic, morphofunctional inference in extinct mammals.

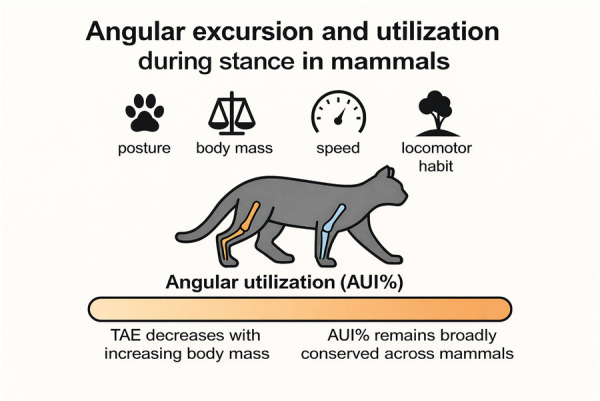

## RESUMEN

Las inferencias morfofuncionales basadas en la estructura anatómica suelen apoyarse en rasgos esqueléticos estáticos, con escasa integración del comportamiento locomotor dinámico. Aunque el movimiento de los miembros de mamíferos exhibe sinergias cinemáticas conservadas, hasta donde sabemos no existe una base de datos comparativa amplia que cuantifique cómo las posturas articulares, las excursiones angulares y la utilización del rango angular varían según factores biológicos. En este estudio se presenta un conjunto comparativo de datos de movimiento articular durante la fase de apoyo de la marcha en 182 especies de mamíferos terrestres que abarcan 15 órdenes, clasificados por postura de los miembros, masa corporal, velocidad máxima de la especie y hábito locomotor. Mediante análisis de video en un plano sagital y fuentes publicadas, se midieron los ángulos articulares en apoyo inicial, medio y despegue para las seis articulaciones principales. A partir de estos datos se calcularon la excursión angular articular (EAA), la excursión angular total (EAT) y un índice de utilización angular (IUA = EAT/∑EAA), expresado como el porcentaje de la excursión articular total (sumada) que se traduce en la excursión neta del miembro durante la fase de apoyo. Mediante mínimos cuadrados generalizados con control filogenético (PGLS) para considerar la no independencia entre especies, la EAA y la EAT mostraron covariación con los factores evaluados, siendo la masa corporal la predictora dominante. La EAT de miembros anteriores y posteriores disminuyó con el aumento de la masa corporal (log_10_), mientras que los efectos de la postura fueron sutiles y presentaron un amplio solapamiento entre categorías. Las especies plantígradas, de pequeño tamaño corporal y arbóreas tendieron a presentar perfiles angulares más amplios, mientras que los taxones ungulígrados, cursoriales y de alta velocidad utilizaron excursiones menores. Las comparaciones basadas en cuadrantes del IUA de miembros anteriores y posteriores resaltaron estrategias locomotoras alineadas con diferentes factores biológicos. En conjunto, estos hallazgos indican que los mamíferos modulan la magnitud y distribución de las excursiones articulares a lo largo de gradientes de tamaño y ecológicos, preservando en gran medida la proporción del rango articular disponible utilizada durante la fase de apoyo, y proporcionando un marco reproducible para interpretar la dinámica de las extremidades en mamíferos actuales y extintos.

## 1 Introduction

Animal movement has been a central theme in biology since the foundational work of Giovanni Alfonso Borelli, who applied physical principles to biological processes, laying the groundwork for iatromechanics (Borelli 1743). Since then, research on locomotion has evolved into a multidisciplinary field encompassing biomechanics, physiology, neuroscience, and evolutionary biology (Alexander 2003; Biewener 2005). Locomotion is critical to a wide range of biological functions, including foraging, predator avoidance, and reproduction (Gatesy and Biewener 1991; Nathan et al. 2008; Dick and Clemente 2016). Understanding how animals move thus provides fundamental insights into functional performance and ecological adaptation.

In tetrapods, evolutionary modifications to the appendicular skeleton have enabled a remarkable diversity of locomotor strategies. Fore‐ and hindlimbs share a common developmental origin in the ancestral chiridium, and detailed comparisons have documented extensive muscular correspondences between them (Owen 1849; Diogo et al. 2008). However, recent analyses have questioned whether these similarities necessarily imply strict serial homology and have emphasized that homology relationships can be context‐dependent (Diogo and Molnar 2014). In this study, I therefore treat fore‐ and hindlimbs as repeated limb modules built on a conserved musculoskeletal blueprint that can be differentially specialized along the body axis. As Lauder (1995) emphasized, a single anatomical design can fulfill multiple functional roles. Likewise, Bock and von Wahlert (1965) proposed that form–function relationships are dynamic, shaped by internal properties (e.g., morphology, physiology) and external pressures (e.g., terrain complexity, predation).

Movement capacity, modulated by biological factors such as body mass, posture, locomotor mode, and speed, is central to the “movement ecology paradigm,” which conceptualizes locomotion as the outcome of interactions between intrinsic traits and extrinsic environmental conditions (Nathan et al. 2008). For example, ground reaction forces (GRFs) are a key environmental influence that modulate musculoskeletal demands (Schmitt 1998), and the evolution of posture and segmental limb design in mammals reflects adaptations to such forces (Lovegrove 2004; Kubo et al. 2019). At the joint level, Pike and Alexander (2002) showed that hindlimb segment proportions are closely related to joint kinematics in quadrupedal mammals, highlighting how morphometric variation constrains and channels angular excursions. This biomechanical perspective aligns with the broader ecological movement framework, which incorporates internal state (e.g., energy reserves, sensorimotor control), movement capacity (e.g., joint mobility), navigation ability, and environmental constraints (Nathan et al. 2008; Joo et al. 2020). Trait‐based approaches increasingly emphasize the importance of linking morphology and physiology to movement outcomes under ecological pressures (Seebacher and Post 2015).

Although pose estimation tools such as DeepLabCut (Mathis et al. 2018) and WildPose (Muramatsu et al. 2025) have advanced field‐based kinematic analysis, these tools remain computationally intensive and not universally accessible. There remains a need for robust, biologically interpretable, and reproducible frameworks for characterizing joint‐level movement in mammals.

The biomechanical significance of joint angular excursion (JAE) is most evident during the stance phase, when limbs are loaded and in contact with the ground. Governed by Newtonian mechanics, this phase encompasses support, braking, and propulsion, and is mechanically coupled to external GRFs (Biewener 2005; Schmitt 1999). Joint excursions during stance therefore provide an integrated readout of internal and external constraints. Moreover, stance mechanics set the kinematic and energetic boundary conditions for the swing phase, influencing subsequent limb trajectories and stride length (Griffin et al. 2004; Pontzer 2007). Contemporary template models emphasize compliant‐leg (SLIP‐like) behavior as a unifying framework across walking and running, with inverted‐pendulum mechanics representing a limiting case for walking (Geyer et al. 2006). Despite this biomechanical importance, relatively few studies have systematically quantified JAEs across species under comparable walking conditions.

Conserved inter‐joint coordination is a hallmark of tetrapod locomotion. Catavitello et al. (2018) demonstrated consistent planar covariation of limb segments across diverse taxa, independent of body size or gait type. Granatosky et al. (2019) further showed that locomotor joints exhibit lower angular variability and higher inter‐joint consistency than feeding joints, underscoring the functional demands of whole‐body propulsion. Similarly, Ross et al. (2013) reported lower stride‐to‐stride variability in mammals and birds compared to reptiles and amphibians, likely reflecting advanced sensorimotor control involving γ‐motoneurons, Ia afferents, and cerebellar integration.

Despite these insights, systematic quantification of joint posture and angular excursion across major biological variables, such as body mass, posture, locomotor habit, and ecological specialization, remains limited. Previous studies have described general coordination patterns, but how these scale with morphological and ecological diversity is still poorly understood. Moreover, although angular regularity has been linked to neuromechanical optimization (Ross et al. 2013; Frigon 2017), few comparative studies have used integrative metrics that assess both joint‐level motion and limb‐level displacement.

To address this, this study poses two central questions: (1) Do JAEs vary systematically with key biological traits across mammals? (2) Is limb‐level angular utilization, as quantified by an angular utilization index (AUI), conserved despite variation in joint excursion? The working hypothesis was that joint excursions would differ across biological categories, reflecting morphofunctional constraints, whereas AUI would be relatively conserved because of shared neuromechanical principles that promote coordinated and stable gait. To test these hypotheses, a comparative data set of JAEs during the stance phase of walking was compiled for 182 species belonging to 15 mammalian orders. This phase, which is both mechanically constrained and functionally informative, provides a standardized window into joint usage. Joint angles and total limb excursion were then examined in relation to limb posture, body size, species maximum speed, and locomotor habit.

An AUI is defined as the ratio between total angular excursion (TAE) and the sum of individual joint excursions (ΣJAE). This biologically interpretable kinematic ratio summarizes how net limb excursion during stance relates to the distribution of joint‐level motion along the limb during loaded locomotion.

Finally, this angular framework provides a conceptual pathway for paleobiological inference by outlining how fossil limb morphologies may be interpreted in relation to extant angular profiles as a complementary, hypothesis‐generating approach.

## 2 Materials and Methods

### 2.1 Animals and Biological Factors

Joint angular data from 182 terrestrial mammal species spanning 15 taxonomic orders were analyzed, focusing on the stance phase during comfortable locomotion. The data set combined original video analyses from publicly available footage (*n* = 81) and supporting data extracted from published literature (*n* = 101; see Supporting Information S1: File 1). All procedures involving video and frame‐sequence analysis were approved by the Institutional Committee for Animal Care and Use at Universidad Católica del Maule (CICUAL‐UCM; Folio No. 10‐2023; 10 May 2023; Talca, Chile).

The influence of four biological factors was evaluated using classification schemes from Medina‐González (2021, 2024) and Vera et al. (2022):

- Body mass was categorized as small (< 1 kg), medium (1–29 kg), large (30–100 kg), or very large (> 100 kg), based on Iriarte‐Díaz (2002), A–Z Animals (2008–2020), Pineda‐Munoz et al. (2016), and Silva and Downing (1995).
- Limb posture was classified as plantigrade, digitigrade, subunguligrade, unguligrade, or mixed (e.g., knuckle‐walking forelimbs with plantigrade hindlimbs), following Carrano (1997).
- Top speed was defined as slow (< 35 km/h), medium (35–50 km/h), or fast (> 50 km/h), per Iriarte‐Díaz (2002).
- Locomotor habits were categorized as cursorial, scansorial, arboreal, or terrestrial based on anatomical and ecological traits (Samuels et al. 2013; Chen and Wilson 2015), Supporting Information S2: File 2.

### 2.2 Data Collection

Joint angles were measured at three key stance‐phase events—touchdown (TD), midstance (MS), and toe‐off (TO)—based on protocols from Fischer et al. (2002), Schilling and Hackert (2006), and Granatosky et al. (2019) (see Supporting Information S3: File 3). These discrete points reflect biomechanically critical transitions in support and propulsion and are compatible with both traditional inverted‐pendulum descriptions and more recent spring‐loaded inverted pendulum (SLIP) models of terrestrial locomotion, in which compliant leg behavior explains the basic dynamics of walking and running (Biewener 2005; Griffin et al. 2004; Geyer et al. 2006). When original video footage was not available for a given species, joint angles and excursions were obtained directly from published studies. Depending on the source, this involved using numerical values reported in results tables, digitizing stance‐phase joint angles from published gait frames, extracting values from summary tables in the supporting material, or analyzing supporting videos linked to the article. The specific source type used for each species is documented in Supporting Information S1: File 1.

**Figure 1:**
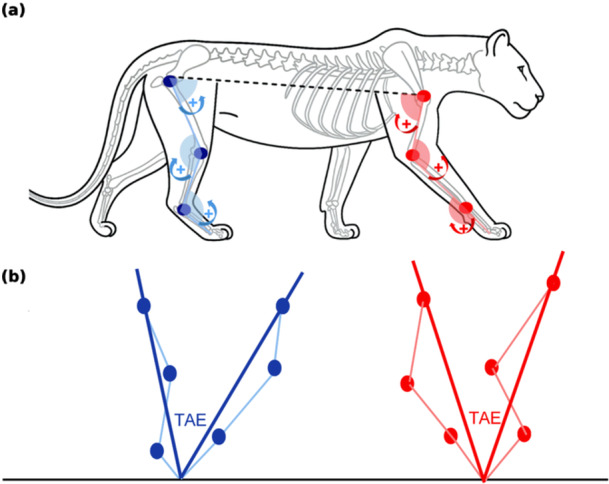
(a) Schematic representation of joint angular excursion (JAE) estimation for forelimb (red) and hindlimb (blue) joints. Angles are calculated for the shoulder, elbow, and wrist in the forelimb, and for the hip, knee, and ankle in the hindlimb. Joint centers were visually estimated based on anatomical landmarks observable in the original data sources (videos and research articles; see Supporting Information S2: File 2 for details). Curved arrows indicate the direction of positive rotation (+), defined as flexion at each joint; extension corresponds to rotation in the opposite direction and is treated as negative (−). (b) Illustration of total angular excursion (TAE), representing the angular path of the functional limb segment between the point of ground contact and the shoulder (forelimb, red) or hip (hindlimb, blue). The TAE trajectory does not pass through individual joint centers, so the sum of joint angular excursions (∑JAE) differs from TAE. This relationship forms the basis of the angular utilization index (AUI), defined as AUI% = (TAE/∑JAE) × 100, which describes the proportion of available joint range recruited during stance.

Videos of walking mammals were sourced online, retaining only sagittal‐plane sequences (30–120 fps) with clear lateral views and sufficient resolution to track anatomical landmarks. Joint centers were visually estimated using external landmarks and species‐specific skeletal models, following a frame‐by‐frame digitizing protocol adapted from Catavitello et al. (2018). Only sequences explicitly described by the original sources as “walking,” “preferred walking speed,” or “comfortable locomotion” were retained, but heterogeneous frame rates and incomplete reporting of spatial calibration prevented consistent reconstruction of absolute or dimensionless speeds for all species. As a result, gait speed was not standardized across taxa and is treated as an inherent component of the functional angular space sampled here. Segmental angles were computed by connecting shoulder, elbow, and wrist (forelimb) and hip, knee, and ankle (hindlimb) markers (Figure 1a).

Angular measurements were obtained using Tracker software (v.4.11.0; Brown 2022), a validated open‐source platform for video motion analysis. This method has shown strong interobserver reliability and criterion validity in previous mammalian gait studies (Medina‐González 2014, 2021, 2024; Vera et al. 2022). Intrarater reliability in this study was excellent (ICC_3,1_ > 0.88), with SEM values below 13°, particularly for the hip, ankle, and shoulder (Supporting Information S4: File 4). These results confirm the reliability and reproducibility of estimating joint kinematics from sagittal videos of diverse terrestrial mammals.

### 2.3 Angular Excursion Analysis

JAE was calculated as the difference between the maximum and minimum angles recorded during the stance phase for each joint. These values were used to build functional angular profiles for each species and biological grouping.

TAE was defined as the angular displacement of the entire limb segment, from the shoulder or hip centroid to the distal contact point, during stance, following Schmidt (2005), Figure 1b.

An AUI was calculated as the ratio between limb‐level TAE and the sum of JAEs across the measured joints for each limb (ΣJAE). AUI% is treated here as a descriptive kinematic ratio that summarizes how net limb excursion during stance relates to the cumulative joint excursions underlying that displacement, and as a proxy for patterns of inter‐joint coordination (Dickinson et al. 2000). The index was computed separately for the forelimb and hindlimb.

$$\text{AUI}\left(\%\right)=\left(\frac{\text{TAE}}{\sum \text{JAE}}\right)\times 100$$

where TAE is the total limb angular excursion during stance and ΣJAE is the sum of JAEs at the shoulder, elbow, and wrist for the forelimb, or at the hip, knee, and ankle for the hindlimb. Higher AUI% values indicate that a greater proportion of the summed joint excursions is expressed as net limb excursion during stance.

### 2.4 Phylogenetic Robustness Analysis of Body Mass and Posture Effects

Phylogenetic comparative methods were used as robustness checks for the primary (nonphylogenetic) analyses. A time‐calibrated mammalian supertree (Upham et al. 2019; DNA‐only maximum clade credibility tree from VertLife) was pruned to the species included in the kinematic data set after standardizing names to *Genus_species*. Size‐related scaling was evaluated using PGLS (*pgls*, caper) under a Brownian‐motion correlation structure (Pagel's *λ* fixed at 1.0), modeling hindlimb and forelimb TAE and AUI% as responses and log_10_ body mass as the predictor. Posture effects on TAE were evaluated using phylogenetic ANOVA (*phylANOVA*, phytools; 1000 simulations; Holm‐corrected pairwise tests). Reproducibility is supported by archiving Supporting Information S3: File 3 (the Excel analysis data set used to run the scripts), together with the R scripts and exported outputs in Zenodo; the input tree is referenced above and can be retrieved from VertLife.

### 2.5 Statistical Analysis

Descriptive statistics were reported as means ± 95% confidence interval (CI). Because reliable absolute or Froude‐scaled speeds could not be obtained for all taxa, gait speed was not included as a covariate; instead, speed‐related variability was treated as an inherent source of ecological and behavioral heterogeneity in the data set rather than as a controlled experimental factor. Group differences in JAE and angular utilization were tested using Welch's ANOVA, followed by Tukey's or Dunnett's T3 post hoc comparisons depending on variance homogeneity. For nonparametric distributions, Kruskal–Wallis tests with Dunn's post hoc corrections were applied. Radar plots were used to summarize raw angular excursion profiles by biological category, displaying mean joint values without scaling.

Associations between limb‐level angular range utilization and biological factors were assessed using scatter plots comparing forelimb (FL) and hindlimb (HL) angular utilization indices (AUI, %), with species classified by category and plotted using standardized color schemes. A quadrant framework, based on 50% AUI thresholds for each limb, was used to identify relative patterns of angular utilization: Quadrant I (FL > 50%, HL > 50%), Quadrant II (FL ≤ 50%, HL > 50%), Quadrant III (FL ≤ 50%, HL ≤ 50%), and Quadrant IV (FL > 50%, HL ≤ 50%). Chi‐squared (*χ* ^2^) tests of independence were applied to assess quadrant distribution by biological factor, and Fisher's exact tests were used when expected cell counts were low.

Pearson correlation coefficients were calculated to examine relationships between TAE and joint‐specific excursions (shoulder, elbow, wrist, hip, knee, ankle), as well as between joint excursions and stride length (normalized for body mass). Pearson correlations were used to assess linear associations, and variables were inspected for outliers and approximate normality prior to analysis. These correlations were stratified by biological category, with results visualized using scatter plots, regression lines with 95% CIs, and tabulated matrices (see Supporting Information S5 and S6: Files 5 and 6).

All statistical tests were performed using GraphPad Prism v6.0 (GraphPad Prism 2012) and IBM SPSS Statistics v20.0. Statistical significance was set at *p* < 0.05.

### 2.6 Use of AI Tools

ChatGPT (OpenAI) was used only to assist with language refinement and with the formatting of schematic/vector figures based on the study's empirical results. No AI tool was used to generate or alter primary data, perform statistical analyses, or produce results. All scientific content, interpretations, and conclusions were critically reviewed, validated, and approved by the author.

## 3 Results

### 3.1 Taxonomic and Biological Diversity

This data set includes 182 terrestrial mammal species from 15 orders, spanning a broad range of body sizes, limb postures, maximum speeds, and locomotor habits. Body mass ranged from 0.09 to 4200 kg (mean = 190.2 kg, 95% CI: 126.4–253.9). Biological‐factor coverage was complete for posture, body mass, and locomotor habit (182/182, 100%), and near‐complete for top speed (179/182, 98%; Supporting Information S3: File 3). This framework informed all analyses of joint poses, angular excursions, total limb displacement, and angular utilization during the stance phase.

### 3.2 Joint Poses and Segmental Angular Contribution

Joint angles recorded at TD, MS, and TO revealed systematic angular transitions (Table 1). In the forelimb, the shoulder angle decreased from 96° ± 21° (TD) to 63° ± 24° (TO), while the elbow angle increased from 127° ± 28° to 142° ± 26°. In the hindlimb, the hip angle rose from 68° ± 27° to 100° ± 27°. JAE was greatest at proximal joints (shoulder: 36° ± 19°, hip: 34° ± 22°), with more even but lower values at distal joints. Relative JAE confirmed that shoulders and hips contributed ~40% of total limb excursion, with distal joints contributing ~30%–36%, indicating a consistent proximal dominance across species (Table 1).

**Table 1:** Joint angles and angular excursion of forelimb and hindlimb joints during the stance phase in terrestrial mammals.

Joint	*n*	TD (°)	MS (°)	TO (°)	JAE (°)	Relative JAE (%)
Shoulder	138	96 ± 21	72 ± 22	63 ± 24	36 ± 19	40.07 ± 16.57
Elbow	151	127 ± 28	129 ± 31	142 ± 26	24 ± 17	26.53 ± 14.01
Wrist	151	181 ± 21	189 ± 30	173 ± 33	35 ± 27	36.33 ± 19.20
Hip	169	68 ± 27	81 ± 26	100 ± 27	34 ± 22	39.88 ± 12.54
Knee	182	128 ± 29	114 ± 28	117 ± 24	26 ± 15	30.69 ± 13.18
Ankle	182	119 ± 34	110 ± 34	127 ± 31	27 ± 15	32.27 ± 14.13

*Note:* Mean (±SD) joint angles measured at touchdown (TD), midstance (MS), and toe‐off (TO) for six major joints across a sample of 182 mammalian species. Joint angular excursion (JAE) is calculated as the difference between the maximum and minimum angle observed during stance. Relative JAE (%) represents the proportional contribution of each joint to the total excursion of its respective limb. Forelimb joints include shoulder, elbow, and wrist; hindlimb joints include hip, knee, and ankle.

### 3.3 Joint Angular Excursion (JAE) and Biological Factors

**Figure 2:**
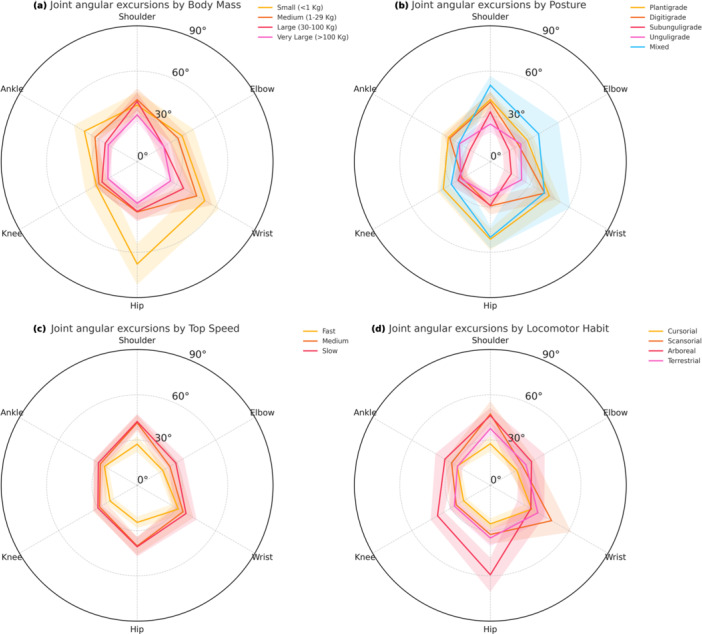
Comparative radar plots of joint angular excursions in terrestrial mammals across biological factors. (a) Joint angular excursions by body mass categories: small (< 1 kg), medium (1–29 kg), large (30–100 kg), and very large (> 100 kg). (b) Joint angular excursions by postural types: plantigrade, digitigrade, subunguligrade, unguligrade, and mixed. (c) Joint angular excursions by maximum speed categories: slow (< 35 km/h), medium (35–50 km/h), and fast (> 50 km/h). (d) Joint angular excursions by locomotor habits: cursorial, scansorial, arboreal, and terrestrial. Each plot displays the mean joint angular excursion (°) for shoulder, elbow, wrist, hip, knee, and ankle during the stance phase of walking. Shaded areas represent the 95% confidence intervals for each biological factor, highlighting variability in joint usage. The radial axis is uniformly scaled from 0° to 90° to encompass variation across all categories. For additional statistical details, see Supporting Information S3: File 3.

Summed joint angular excursions (∑JAE) per limb differed significantly across biological categories (Table 2), as illustrated by the angular profiles in Figure 2.

**Table 2:** Total angular excursion and angular range utilization of forelimbs and hindlimbs according to biological factors.

Biological factors		Forelimb			Hindlimb		
		TAE (°)	∑ JAE (°)	AUI (%)	TAE (°)	∑ JAE (°)	AUI (%)
Posture	Plantigrade	64 ± 19^a^ (22)	115 ± 45^a^ (37)	56.4 ± 19.7^a,b^ (22)	67 ± 15^a^ (31)	119 ± 45^a^ (47)	51.9 ± 16.2^a^ (32)
	Digitigrade	57 ± 12^a,b^ (28)	95 ± 52^a,b^ (44)	53.3 ± 12.2^b^ (28)	50 ± 12^b^ (31)	82 ± 39^b^ (47)	57.0 ± 14.2^a^ (32)
	Subunguligrade	45 ± 4^b^ (7)	63 ± 10^c^ (12)	74.5 ± 12.3^a^ (7)	42 ± 6^b,c^ (7)	69 ± 14^b, c^ (12)	63.8 ± 14.8^a^ (7)
	Unguligrade	47 ± 12^b^ (30)	77 ± 27^c^ (30)	65.2 ± 15.3^a^ (30)	40 ± 8^c^ (30)	69 ± 21^b,c^ (58)	56.1 ± 16.2^a^ (30)
	Mix	47 ± 13^a,b^ (6)	129 ± 48^a^ (12)	38.9 ± 14.9^c^ (6)	52 ± 17^b,c^ (6)	104 ± 31^a,c^ (12)	59.2 ± 18.6^a^ (6)
Body mass	Small	77 ± 17^a^ (8)	124 ± 44^a^ (15)	65.3 ± 21.9^a^ (8)	78 ± 9^a^ (15)	139 ± 56^a^ (17)	56.4 ± 14.1^a^ (15)
	Medium	60 ± 17^b^ (21)	113 ± 58^a,b^ (38)	53.8 ± 19.3^a^ (21)	54 ± 13^b^ (23)	95 ± 32^b^ (57)	50.2 ± 17.0^a^ (24)
	Large	52 ± 13^b,c^ (20)	92 ± 45^b,c^ (29)	54.7 ± 17.9^a^ (20)	49 ± 15^b^ (20)	84 ± 42^b,c^ (39)	58.4 ± 14.7^a^ (20)
	Very large	48 ± 10^c^ (44)	79 ± 28^c^ (53)	61.3 ± 14.3^a^ (44)	42 ± 9^c^ (44)	72 ± 25^c^ (69)	57.4 ± 15.8^a^ (44)
Top speed (species maximum speed)	Slow	61 ± 20^a^ (25)	111 ± 49^a^ (44)	56.9 ± 20.0^a^ (25)	62 ± 19^a^ (33)	100 ± 45^a^ (66)	54.8 ± 14.9^a^ (33)
	Average (med)	57 ± 12^a,b^ (27)	98 ± 41^a,b^ (36)	55.2 ± 14.9^a^ (27)	51 ± 12^b^ (28)	97 ± 38^a^ (43)	54.8 ± 13.1^a^ (28)
	Fast	47 ± 11^b^ (40)	79 ± 38^b^ (52)	61.4 ± 16.6^a^ (40)	43 ± 10^c^ (40)	71 ± 28^b^ (70)	58.5 ± 15.9^a^ (40)
Locomotor habit	Cursorial	48 ± 10^a^ (39)	80 ± 38^a^ (50)	62.8 ± 16.3^a^ (39)	43 ± 11^a^ (39)	71 ± 25^a^ (68)	56.4 ± 13.7^a^ (39)
	Scansorial	61 ± 16^b^ (17)	113 ± 65^b^ (23)	54.6 ± 21.1^a^ (17)	55 ± 14^b^ (19)	88 ± 39^a,b^ (38)	61.7 ± 15.4^a^ (19)
	Arboreal	78 ± 21^c^ (6)	106 ± 48^a,b^ (18)	67.3 ± 15.0^a^ (6)	79 ± 6^c^ (12)	134 ± 50^c^ (25)	49.0 ± 11.3^a^ (12)
	Terrestrial	53 ± 13^a,b^ (31)	102 ± 38^a,b^ (44)	53.6 ± 14.9^a^ (31)	50 ± 15^b^ (32)	88 ± 33^a,b^ (51)	54.0 ± 18.7^a^ (33)

*Note:* Mean values (±SD) of total angular excursion (TAE), summed joint angular excursion (∑JAE), and angular utilization index (AUI%; [TAE/∑JAE] × 100) for forelimbs and hindlimbs of terrestrial mammals, grouped by posture, body mass, top speed (species maximum speed), and locomotor habit. Superscript letters indicate significant differences between categories within each column (Tukey's post hoc, *p* < 0.05). Sample sizes (*n*) per group are shown in parentheses.

Body mass was negatively associated with JAE (Table 2 and Figure 2a). Small mammals exhibited greater angular displacements in both forelimbs (124° ± 44°) and hindlimbs (139° ± 56°) compared to very large mammals (forelimb: 79°± 28°; hindlimb: 72° ± 25°). This trend was especially pronounced at the hip and shoulder, reflecting the greater mobility of proximal joints in smaller‐bodied species. The radar plot shows a radial contraction in joint angles with increasing size, particularly in distal joints such as the wrist and ankle.

Limb posture also influenced total JAE values (Figure 2b). In the forelimb, plantigrade species displayed the highest JAE (115°± 45°), significantly exceeding those of subunguligrade (63°± 10°) and unguligrade species (77° ± 27°). Similarly, plantigrades showed greater hindlimb JAE (119° ± 45°), whereas unguligrades and subunguligrades were comparably lower (~69°). The radar plot indicates that digitigrades exhibited intermediate values, while mixed‐posture species showed disproportionately high excursions at specific joints such as the wrist and hip.

Top speed (species maximum speed) was inversely related to angular excursion (Table 2 and Figure 2c). Species classified as slow movers presented broader JAE in both forelimbs (111° ± 49°) and hindlimbs (100° ± 45°), compared to fast‐moving taxa (forelimb: 79° ± 38°; hindlimb: 71° ± 28°). This reduction was particularly evident at the wrist and ankle, suggesting a kinematic constraint associated with high‐speed locomotion.

Locomotor habit was also a key determinant of JAE patterns (Figure 2d). Arboreal mammals exhibited the greatest hindlimb JAE (134°± 50°), whereas scansorial species displayed the highest values in the forelimb (113° ± 65°). Cursorial species had the most constrained joint profiles, with forelimb excursions averaging 80° ± 38° and hindlimb excursions 71° ± 25°. These differences reflect biomechanical demands associated with substrate complexity and movement versatility. Radar plots emphasize the distributed joint usage in arboreal taxa versus the more compact angular profiles of terrestrial runners.

Collectively, these patterns indicate that JAE is modulated by biological factors in a region‐specific manner: proximal joints (shoulder, hip) account for larger differences across body mass and posture categories, while distal joints (wrist, ankle) show more pronounced variation in relation to speed and ecological niche.

### 3.4 Total Angular Excursion (TAE)

TAE, the cumulative angular displacement across the limb during stance, declined significantly with increasing body mass (forelimb ANOVA *p* = 0.004, hindlimb *p* = 0.002) and was highest in plantigrades (forelimb: 64° ± 19°, hindlimb: 67° ± 15°). Subunguligrades and unguligrades exhibited markedly lower TAE values (e.g., forelimb: ~45°–47°, hindlimb: ~40°–42°). Similarly, arboreal and scansorial mammals showed elevated TAE (up to 79°), while cursorial taxa displayed the lowest values (forelimb: 48° ± 10°, hindlimb: 43° ± 11°), suggesting ecological tuning of angular use (Table 2).

### 3.5 Angular Utilization Index (AUI%)

The angular utilization index (AUI% = TAE/∑JAE) was highest in subunguligrade and unguligrade forelimbs (74.5% and 65.2%), and lowest in mixed‐posture species (38.9%). In the hindlimb, AUI showed minimal postural differences (*p* = 0.388) but was numerically higher in unguligrades (63.8%) than plantigrades (51.9%). Scansorial mammals displayed the highest hindlimb AUI (61.7%), significantly exceeding arboreal species (49.0%, *p* = 0.041), suggesting more concentrated use of available joint range during stance in taxa adapted to complex substrates. Forelimb AUI was moderately related to speed (*p* = 0.022), with fast taxa showing the highest values (64.7%), Table 2.

### 3.6 Correlations Between Excursion, Body Mass, and Stride Length

**Figure 3:**
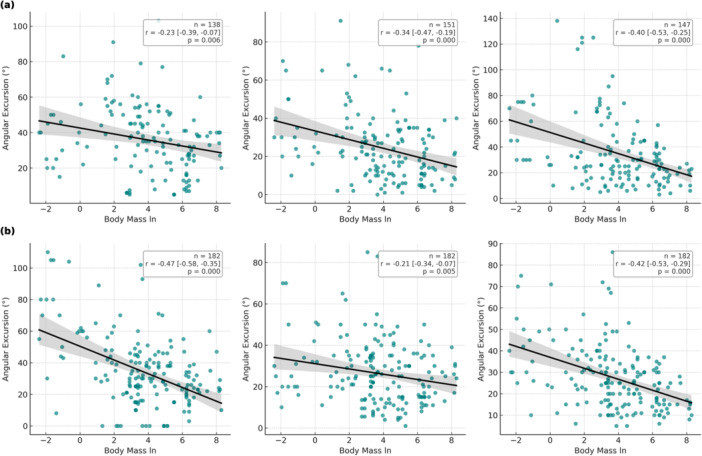
Scatter plots showing the relationship between body mass (log‐transformed, ln) and joint angular excursion (°) during stance in mammals. Each panel represents a specific joint: shoulder, elbow, wrist (a); and hip, knee, ankle (b). Regression lines with 95% confidence intervals are shown. For each plot, the sample size (*n*), Pearson's correlation coefficient (*r*) with 95% confidence interval, and the corresponding *p* value are provided. Note that joint‐specific sample sizes vary due to missing data.

Joint excursion showed strong negative correlations with body mass across several joints (Figure 3). In the forelimb, wrist excursion had the strongest inverse correlation (*r* = –0.40), followed by elbow (*r* = –0.34) and shoulder (*r* = –0.23). In the hindlimb, the hip (*r* = –0.47) and ankle (*r* = –0.42) showed the steepest declines.

**Figure 4:**
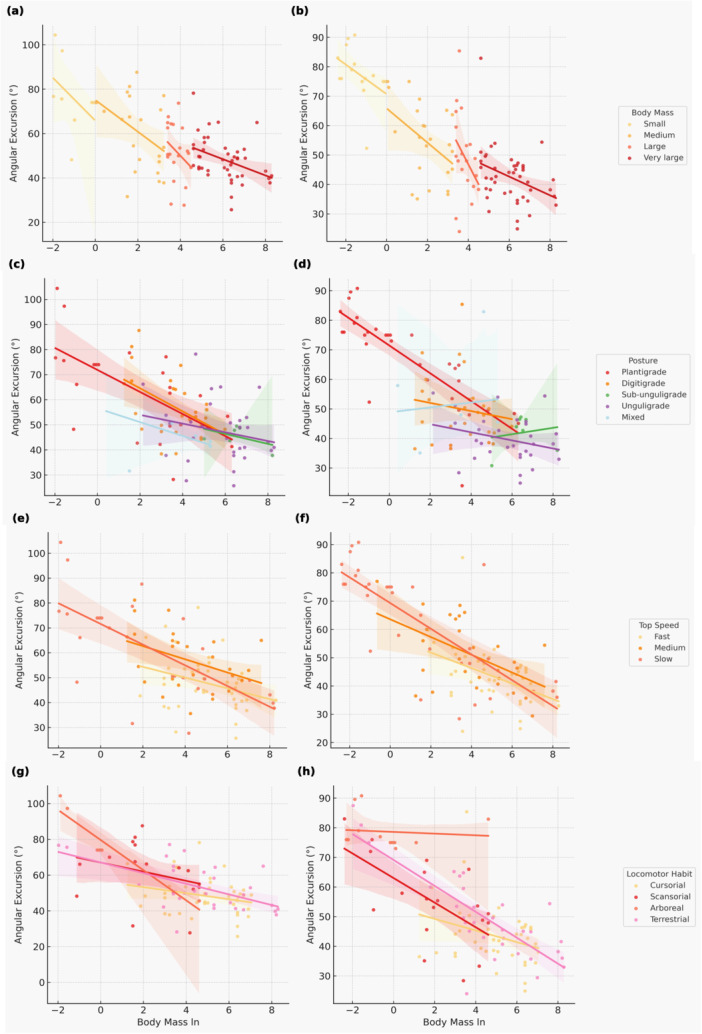
Scatter plots showing the relationship between log‐transformed body mass (ln Body Mass) and total angular excursion (TAE) during the stance phase in mammals. Forelimb data are presented in (a), (c), (e), and (g); hindlimb data are shown in (b), (d), (f), and (h). Each point represents a species, with regression lines and 95% confidence intervals. (a and b) Species categorized by body mass: small (yellow), medium (orange), large (light red), very large (dark red). (c and d) Species categorized by limb posture: plantigrade (red), digitigrade (orange), subunguligrade (green), unguligrade (purple), mixed (light blue). (e and f) Species categorized by maximum top speed: fast (yellow), medium (orange), slow (red). (g and h) Species categorized by locomotor habit: cursorial (yellow), scansorial (red), arboreal (pink), terrestrial (light pink). For additional statistical details, see Supporting Information S5: File 5.

At the limb level, TAE was inversely related to body mass (Figure 4). These scaling trends were especially pronounced in plantigrades (hip *r* = –0.57, elbow *r* = –0.41, ankle *r* = –0.44), Figure 4 and Supporting Information S5: File 5.

Contrary to expectations, JAE was not positively associated with stride length at the whole‐sample level. As shown in Supporting Information S8: Figure 1, correlations between angular displacement and stride length were generally weak or negative. The wrist exhibited the strongest negative association (*r* = –0.40, *p* < 0.001), followed by the ankle (*r* = –0.42, *p* < 0.001), indicating that larger angular excursions at distal joints were associated with shorter stride lengths. Similar but weaker trends were observed at the elbow (*r* = –0.34, *p* < 0.001) and hip (*r =* –0.47, *p* < 0.001), while shoulder and knee excursions showed no significant associations.

Stratified analyses by body size provided additional insights. In small mammals, wrist excursion was negatively correlated with mass‐normalized stride length (*r* = –0.59, *p* = 0.022), suggesting a potential stabilization‐related pattern during gait. Among medium‐sized species, hip excursion remained the only joint significantly correlated with normalized stride length (*r* = 0.54, *p* = 0.018). No significant joint–stride length associations were found in large‐bodied mammals, indicating a shift toward alternative locomotor strategies where stride performance is governed by limb posture and inertial constraints rather than angular range alone.

### 3.7 Phylogenetic Validation of Size‐ and Posture‐Related Angular Patterns

Phylogenetic analyses were conducted as robustness checks of the primary results. Under a Brownian‐motion correlation structure (Pagel's *λ* fixed at 1.0), PGLS revealed significant negative scaling of TAE with log₁₀ body mass in both limbs (hindlimb: slope = −10.33 ± 2.54°, *t*_52_ = −4.06, *p* < 0.001, *R* ^2^ = 0.24; forelimb: slope = −10.62° ± 3.79°, *t*_44_ = −2.80, *p* = 0.0075, *R* ^2^ = 0.15). In contrast, AUI showed no significant mass dependence (hindlimb: slope = 0.040 ± 0.048, *p* = 0.41, *R* ^2^ = 0.01; forelimb: slope = −0.11 ± 0.07, *p* = 0.12, *R* ^2^ = 0.05), indicating broadly conserved angular utilization across body sizes despite reduced absolute excursions in larger mammals. Phylogenetic ANOVA detected no significant posture effects on TAE (hindlimb: *F* = 7.28, *p* = 0.12; forelimb: *F* = 1.88, *p* = 0.71; 1000 simulations; Holm‐corrected pairwise *p* ≥ 0.48), consistent with subtle and overlapping postural differences in a phylogenetic context.

### 3.8 Fore–Hindlimb AUI Quadrant Analysis

**Figure 5:**
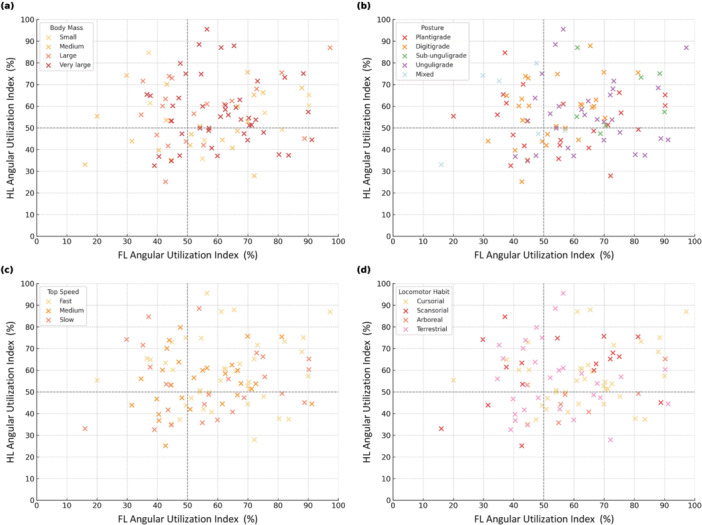
Scatter plots show the relationship between forelimb (FL) and hindlimb (HL) angular range utilization (AUI, %) in mammalian species. Dotted lines at 50% AUI delineate four quadrants representing combined patterns of FL and HL usage: Quadrant I (FL > 50%, HL > 50%), Quadrant II (FL ≤ 50%, HL > 50%), Quadrant III (FL ≤ 50%, HL ≤ 50%), and Quadrant IV (FL > 50%, HL ≤ 50%). Each point represents a species, and biological categories are color‐coded. Sample sizes vary across factors; detailed counts are provided in Supporting Information S7: File 7. (a) Species categorized by body mass: small (yellow), medium (orange), large (light red), very large (dark red). (b) Species categorized by limb posture: plantigrade (red), digitigrade (orange), subunguligrade (green), unguligrade (purple), mixed (light blue). (c) Species categorized by top speed: fast (yellow), medium (orange), slow (red). (d) Species categorized by locomotor habit: cursorial (yellow), scansorial (red), arboreal (pink), terrestrial (light pink).

To assess limb coordination patterns, species were classified into four AUI quadrants based on whether their forelimb (FL) and hindlimb (HL) angular utilization index (AUI%) values were above or below a 50% threshold. Quadrant distributions are shown in Figure 5, and full species classifications are provided in Supporting Information S7: File 7.

*χ* ^2^ tests indicated no overall association between body mass and quadrant distribution (*χ* ^2^ = 12.59, df = 9, *p* = 0.182), but Fisher's exact tests revealed specific patterns: very large species were significantly overrepresented in Quadrant I (*p* = 0.015), indicating high and coordinated angular range utilization in both limbs, whereas medium‐bodied species were more frequent in Quadrant IV (*p* = 0.021), consistent with a forelimb‐dominant AUI profile.

Limb posture showed a marginal association with quadrant distribution (*χ* ^2^ = 19.93, df = 12, *p* = 0.068), with subunguligrade species significantly clustered in Quadrant I (*p* = 0.0198), reflecting coordinated high AUI in both fore‐ and hindlimbs.

Species maximum speed was significantly related to quadrant membership (*χ* ^2^ = 14.26, df = 6, *p* = 0.027). Slow‐moving species were overrepresented in Quadrant IV (*p* = 0.0029) and underrepresented in Quadrant I (*p* = 0.0078), suggesting a pattern in which forelimb joints recruit a larger fraction of their summed joint excursions than hindlimb joints. Medium‐speed taxa also showed a tendency toward Quadrant IV (*p* = 0.032).

Locomotor habit was also significantly associated with AUI quadrant (*χ* ^2^ = 22.98, df = 9, *p* = 0.006). Arboreal species were highly concentrated in Quadrant IV (*p* = 0.00004), supporting the notion that forelimb AUI dominates in taxa adapted to complex substrates. Cursorial mammals, by contrast, were overrepresented in Quadrant I (*p* = 0.038), consistent with more balanced and coordinated limb mechanics. Terrestrial mammals showed a borderline association with Quadrant III (*p* = 0.050), where both fore‐ and hindlimbs exhibited relatively low AUI.

## 4 Discussion

Understanding how mammals use their limbs during the stance phase of walking provides key insight into the intersection of form, biomechanics, and ecological adaptation. Across 182 species, joint‐ and limb‐level excursions (JAE and TAE) varied systematically with body mass, posture, maximum speed category, and locomotor habit, whereas angular utilization (AUI%) remained comparatively stable, particularly in the hindlimb. Phylogenetic analyses used as robustness checks supported these patterns: stance‐phase TAE scaled negatively with body mass in both limbs, AUI% showed little evidence of mass dependence, and posture effects on TAE were subtle and strongly overlapping after accounting for shared ancestry. Together, the data set is best interpreted as a descriptive framework of stance‐phase angular use across mammals, rather than as speed‐standardized gait comparisons.

### 4.1 Joint Pose and Segmental Limb Excursion Across Mammals

Joint pose data revealed consistent interjoint coordination across the stance phase, with predictable angular transitions at TD, MS, and TO. In both forelimbs and hindlimbs, the shoulder and hip exhibited the greatest angular displacement, while the elbow, knee, wrist, and ankle showed more moderate excursions (Table 1). These findings highlight the dominant mechanical role of proximal segments in driving limb motion during support and align with established principles of limb segment dynamics, where proximal joints contribute the bulk of net positive mechanical work during stance (Winter 2009; Biewener and Daley 2007). This pattern has been confirmed across vertebrates through both empirical and simulation‐based studies, from avian bipeds to quadrupedal mammals (Roberts and Scales 2002; Biewener and Daley 2007), supporting the interpretation that preferential recruitment of proximal joint ranges reflects a conserved strategy for force generation and energy transfer.

The values reported represent general trends across a taxonomically and morphologically diverse sample of terrestrial mammals, encompassing a broad range of body sizes, postural strategies, and locomotor behaviors. Accordingly, joint poses and excursions are expected to vary with biological factors. Mammalian locomotor patterns are shaped by phylogeny, body mass, and ecological context, all of which influence limb posture, joint range, and gait strategy (Carrano 1997; Jenkins 1971; Hutchinson 2021). While the data set outlines overarching patterns, stratified analyses are essential for revealing functional differences within this diversity.

### 4.2 Angular Excursion Across Biological Factors

JAE and TAE showed marked differences across body mass, speed, and locomotor habits. Small mammals consistently displayed greater total JAE and TAE in both limbs, which likely reflects increased joint flexibility and maneuverability. In contrast, large‐bodied species showed constrained joint motion, particularly at proximal joints, aligning with biomechanical models that predict reduced excursion to preserve structural integrity and minimize energetic cost (Alexander et al. 1979; Biewener 1989a, 1989b). However, after accounting for shared ancestry, posture explained little additional variation in TAE, with extensive overlap among postural categories, suggesting that broad postural labels capture only part of the functional signal and may partly reflect clade‐level clustering rather than discrete locomotor regimes. In the nonphylogenetic patterns, arboreal and plantigrade taxa tended to show broader excursions than cursorial and unguligrade taxa (Figure 2), highlighting the influence of ecological context and substrate complexity on joint‐range deployment (Cartmill 1985; Clemente et al. 2019).

Maximum speed category was inversely associated with angular excursion: fast‐associated taxa used smaller excursions while maintaining relatively high AUI%, particularly in cursorial forms, consistent with straighter limb postures and reduced joint excursions at higher locomotor demands (Hildebrand 1989; Garland and Janis 1993). In mechanistic terms, these patterns align with compliant‐leg (SLIP‐like) dynamics, with inverted‐pendulum mechanics representing a limiting case for walking (Geyer et al. 2006; Biewener 2005), whereas arboreal and scansorial taxa prioritize broader angular mobility for negotiating complex substrates.

### 4.3 Angular Range Utilization and Functional Constraint

Despite substantial variation in JAE across species, angular range utilization during stance remained relatively stable, especially in the hindlimb. This consistency across a broad range of mammalian morphologies and ecological strategies suggests that limb coordination is governed by underlying neuromechanical constraints—principles that stabilize locomotor output regardless of structural diversity (Catavitello et al. 2018; Frigon 2017). These constraints emerge from the integration of spinal central pattern generators, sensory feedback, and descending control pathways, which together produce repeatable patterns of joint recruitment while walking.

Quadrant‐based analysis using a 50% angular utilization threshold for both forelimb and hindlimb revealed biologically meaningful patterns. Species in Quadrant I, with AUI values above 50% in both limbs, included many large‐bodied and cursorial mammals. These forms likely benefit from coordinated stance‐phase limb use that minimizes muscular effort through straighter limb postures and reduced angular displacement. In contrast, species in Quadrant IV, with higher forelimb but lower hindlimb AUI, were predominantly arboreal and slow‐moving. This asymmetry likely reflects a functional shift toward forelimb‐dominant coordination in animals that navigate complex or variable substrates.

The consistency of hindlimb angular utilization across biological factors supports its conserved role in propulsion and mechanical work generation. This pattern aligns with biomechanical studies showing that hindlimb mass properties and joint moments scale predictably with body size (Kilbourne and Hoffman 2013). It is also consistent with the neuroanatomical organization of mammalian locomotor circuits, in which hindlimb control is more strongly associated with rhythm generation and interlimb coordination (Frigon 2017).

In contrast, forelimb angular utilization was more variable, particularly in taxa where the forelimbs contribute substantially to braking, balancing, or substrate interaction. In many mammals, the forelimbs tend to absorb a larger share of decelerating GRFs during stance, whereas the hindlimbs contribute more to net propulsion. Any suggestion that particular clades emphasize forelimb braking should therefore be treated as a working hypothesis and would require direct ground‐reaction force data, which are beyond the scope of this kinematic data set. This variability likely reflects the multifunctional role of the forelimb in navigating heterogeneous terrains (Fabre et al. 2015). Neural control systems may accommodate this flexibility through modular organization, allowing independent modulation of forelimb and hindlimb outputs. Propriospinal circuits and reflexive pathways enable forelimb‐specific adaptations without disrupting overall gait coordination.

From a neuromechanical standpoint, the conservation of hindlimb angular utilization likely reflects the action of central pattern generators and muscle synergies that stabilize movement while minimizing energetic cost. Evidence from human and animal studies shows that shared muscle activation patterns are used across tasks such as walking and balance control, supporting the idea of conserved motor modules that simplify control and constrain variation (Chvatal and Ting 2013).

Together, these findings indicate that while joint excursions are modulated by ecological and morphological demands, angular range utilization appears constrained by evolutionary principles that promote coordinated and economical limb function. This pattern aligns with predictive musculoskeletal models that relate posture, body mass, and metabolic cost (Clemente et al. 2024) and is consistent with the self‐stabilizing architecture of trisegmented limbs in therian mammals. As Fischer and Blickhan (2006) emphasized, the proximal dominance in propulsion and distal modulation for postural control underpin a neuromechanically constrained locomotor strategy that is conserved across ecological and phylogenetic diversity.

This supports a broader interpretation in which mammalian limb mechanics reflect both adaptive versatility and deeply embedded neuromechanical organization.

### 4.4 Excursion Dynamics and Locomotor Output

Scaling analyses revealed a consistent decline in JAE with increasing body mass (Figures 3 and 4). Distal joints showed the strongest negative associations with log‐transformed mass, particularly the wrist (*r* = −0.40) and ankle (*r* = −0.42), and the hip also exhibited a pronounced scaling effect (*r* = −0.47), especially among plantigrade and medium‐sized taxa (Supporting Information S5: File 5). Phylogenetic robustness checks supported these trends: in PGLS models, hindlimb and forelimb TAE decreased by ~10° per unit increase in log_10_ body mass, whereas AUI% showed little evidence of mass dependence, indicating that larger mammals reduce absolute stance‐phase excursions while maintaining broadly similar angular utilization ratios across body sizes (see Section 3; Kilbourne and Hoffman 2013).

These scaling patterns are consistent with increasing inertial and mechanical constraints on limb mobility as body size increases. In the forelimb, reduced excursions align with the positive allometry of moment of inertia (∝M^1.78^), which limits angular acceleration capacity (Kilbourne and Hoffman 2013), whereas reduced hindlimb excursion may also reflect functional demands of load support and postural economy. Evidence from large mammals further suggests that reduced limb angular displacement can be compensated by alternative strategies, including straighter limb alignments and more pendular mechanics, to maintain stride length with minimal muscular effort (Hutchinson 2021).

Contrary to initial expectations, JAE was not positively associated with stride length at the whole‐sample level (Supporting Information S8: Figure 1). Distal joint excursions (wrist and ankle) showed significant negative correlations with absolute stride length, while shoulder and knee excursions were not significantly related. Stratified analyses indicated that only a limited subset of associations persisted within size classes (e.g., a negative wrist–normalized stride‐length relationship in small mammals and a positive hip‐normalized stride‐length relationship in medium‐sized mammals; Supporting Information S6: File 6), and no significant joint–stride‐length correlations were detected in large‐bodied taxa.

Together, these results indicate that, within this comparative data set, longer strides and higher maximum speeds are not explained simply by larger stance‐phase joint excursions. Instead, variation in locomotor output is consistent with combined changes in stride length and stride frequency and with other mechanical mechanisms that affect effective limb length and whole‐body displacement (e.g., more extended limb postures, scapular and pelvic contributions, and elastic energy storage and recovery in distal tendons; Alexander 1989; Biewener and Daley 2007; Reilly et al. 2007).

The negative distal correlations are consistent with a stabilizing role of the wrist and ankle during stance, emphasizing control and substrate interaction rather than propulsion (Alexander 1989; Griffin et al. 2004). Because scapular motion was not directly quantified, forelimb excursion estimates likely remain conservative in taxa where scapular contributions are substantial, particularly in larger cursorial mammals. Overall, the results support a functional partitioning in which distal joints contribute primarily to stability and precision, while proximal joints are more directly involved in stride modulation, with size‐related mechanical constraints limiting the extent to which joint excursions alone can explain variation in stride length (Kilbourne and Hoffman 2013).

### 4.5 Evolutionary and Comparative Implications

Across terrestrial mammals, hindlimb angular utilization (AUI%) was comparatively conserved despite substantial variation in absolute joint and limb excursions, consistent with stabilizing neuromechanical constraints on locomotor coordination (Catavitello et al. 2018; Frigon 2017). This conservation was most evident in the hindlimb, where joint function remains tightly coupled to support and propulsion across body sizes and ecological contexts. In contrast, forelimb AUI% was more variable, consistent with the modular role of the forelimb in braking, balancing, and substrate interaction, and with the pronounced functional disparity of mammalian forelimbs across lineages. This contrast is consistent with comparative evidence that locomotor joints tend to show stronger kinematic consistency than joints used in other functional contexts, reflecting constraints related to effective force production during gait (Granatosky et al. 2019). This pattern contrasts with other cyclical systems (e.g., feeding), which operate under different mechanical constraints. Locomotor function in extinct megafauna likely involved distinctive trade‐offs among mass support, stability, and performance that can complicate direct modern analogies (Hutchinson 2021).

By integrating angular metrics with posture, body mass, species maximum speed (top speed), and locomotor habit, this data set provides a reproducible comparative framework for interpreting stance‐phase limb mechanics in living taxa and generating testable hypotheses for extinct mammals. A key contribution is the quadrant‐based analysis of forelimb and hindlimb AUI% (Figure 5), which summarizes coordinated versus asymmetric limb‐level utilization patterns in a simple, interpretable classification. This framework can be extended to fossil taxa by placing predicted AUI% combinations into quadrants to evaluate plausible locomotor “solutions,” such as coordinated high utilization in both limbs versus forelimb‐dominant utilization consistent with maneuvering on complex substrates. Used conservatively, quadrant assignments offer a tractable way to compare extinct forms against extant functional distributions and to bound expectations about limb coordination and stance‐phase joint recruitment when soft tissues and direct kinematics are unavailable.

These applications should be interpreted with appropriate caution. Phylogenetic analyses indicate that posture‐related differences in stance‐phase TAE are subtle and strongly overlapping once shared ancestry is considered, and that size‐related scaling trends, although statistically significant, are relatively weak. Accordingly, angular ranges from this data set are best treated as broad, phylogenetically structured envelopes rather than precise predictions for individual fossil species, and projections are most defensible when evaluated within appropriate clades and alongside independent anatomical and biomechanical evidence (Upham et al. 2019).

Within this context, morphofunctional space approaches (Medina‐González 2021; Vera et al. 2022; Medina‐González and Moreno 2025) offer a structured way to situate extinct forms within biomechanical and ecological axes informed by living taxa. The present data set expands that toolkit by adding stance‐phase angular profiles and utilization ratios as kinematic constraints that complement established proxies such as muscle reconstructions and effective mechanical advantage. This framework has been illustrated in a recent case study of Miocene mesotheriid notoungulates, where stance‐phase angular ranges were estimated by combining astragalar morphometrics with the comparative mammalian data set (Medina‐González 2025). More broadly, consistent with the view that locomotor performance reflects trade‐offs among support, stability, and energetic economy (Hutchinson 2021), the comparatively conserved hindlimb AUI% suggests that aspects of neuromechanical organization may constrain limb‐level solutions even as morphology diversifies. This aligns with the perspective that form can delimit feasible ranges while neural coordination influences how those ranges are recruited during stable regimes of joint use (Lauder 1995).

### 4.6 Limitations and Future Directions

This study used a two‐dimensional sagittal‐plane approach to quantify stance‐phase JAEs during comfortable walking. While this procedure supports reproducible cross‐species comparisons, several limitations should be considered.

First, gait speed was not standardized across species. Although sequences were selected as comfortable or preferred walking in the original sources, the underlying video material varied in temporal resolution and often lacked the information required to derive reliable absolute speeds for every taxon. Thus, speed‐related differences could not be incorporated as an explicit covariate and likely contribute to residual scatter in several relationships. Future work using standardized speed measurements, or independent data sets with comparable kinematic sampling, would help refine these scaling estimates. In addition, angular measurements were derived from three discrete time points—TD, MS, and TO—rather than from continuous motion tracking. Nonetheless, the resulting excursion values fall within expected ranges reported in previous studies, supporting both the reliability and concurrent validity of the approach (Granatosky et al. 2019; Catavitello et al. 2018; see Supporting Information S4: File 4).

Second, several relationships show substantial interspecific scatter (e.g., Figures 3 and 4). Many trends are statistically significant but weak, indicating that a large proportion of variance is not explained by the predictors considered. Accordingly, angular ranges derived here should be treated as broad probabilistic envelopes rather than precise predictions for individual species, particularly when extrapolating to extinct taxa. A formal predictive validation, for example, testing how well these envelopes recover joint‐level kinematics in extant mammals withheld from model fitting, is an important direction for future research. Third, phylogenetic comparative analyses were implemented as robustness checks, but PGLS models were fitted under a conservative Brownian‐motion structure with Pagel's *λ* fixed at 1.0. Estimating *λ* and evaluating alternative correlation structures could refine phylogenetic effect sizes in future work. In addition, phylogenetic ANOVAs indicated subtle and strongly overlapping posture‐related differences in stance‐phase TAE once shared ancestry is considered, which cautions against interpreting posture categories as discrete functional regimes.

Fourth, forelimb kinematics do not incorporate scapular motion. Because scapular translation and rotation can contribute substantially to limb excursion, forelimb angular profiles likely represent conservative estimates in taxa with high shoulder mobility, especially cursorial and scansorial mammals. This limitation may dampen limb‐level contrasts between forelimb and hindlimb metrics and should be addressed in future data sets that quantify scapulothoracic contributions explicitly.

Finally, the data set does not explicitly separate gait types (e.g., symmetrical walking vs. asymmetrical gaits), and richer multimodal data would strengthen mechanistic interpretation. Integrating electromyography, energetic cost, GRFs, and substrate properties would help link angular utilization to underlying neuromechanical and mechanical drivers. In parallel, markerless pose estimation tools, including DeepLabCut (Mathis et al. 2018) and WildPose (Muramatsu et al. 2025), offer promising avenues for expanding comparable kinematic data sets under naturalistic conditions where continuous motion capture is not feasible.

## 5 Conclusions

This study quantifies stance‐phase joint angular displacement in terrestrial mammals and evaluates how it varies with key biological factors. JAE and TAE differed systematically across body mass, posture, species maximum speed, and locomotor habit, consistent with broad scaling and eco‐morphological effects on limb kinematics. In contrast, angular range utilization (AUI%), expressed as the proportion of summed joint excursions that contributes to net limb excursion during stance, was comparatively stable in the hindlimb across taxa, while the forelimb showed greater variability. Together, these patterns are consistent with a locomotor organization in which mammals adjust the absolute magnitude of joint excursions across size and ecological gradients while maintaining a relatively constrained distribution of joint use during stance.

Scaling analyses indicated that joint and limb excursions generally decline with increasing body mass, and joint‐specific correlations suggest that distal joints are especially sensitive to size‐ and speed‐related constraints. Stride length was not amplified by larger joint excursions at the whole‐sample level, and associations were weak and context‐dependent, supporting the view that mammals can achieve stride modulation through multiple strategies beyond increasing angular displacement alone. The quadrant‐based analysis of forelimb and hindlimb AUI% provides a simple comparative classification of coordinated versus asymmetric limb‐level utilization patterns that can be used to generate testable hypotheses about limb coordination in taxa with limited kinematic information.

By embedding these angular metrics in a comparative morphofunctional framework, this data set offers a reproducible baseline for describing stance‐phase limb mechanics in extant mammals and for constraining probabilistic kinematic envelopes relevant to paleobiological inference. When integrated with anatomical proxies and phylogenetic context, joint excursion profiles and AUI% can help bound plausible locomotor scenarios for extinct taxa and support hypothesis‐driven reconstructions of stance‐phase function. Used cautiously as probabilistic envelopes rather than point predictions, these angular profiles can complement established anatomical proxies to evaluate competing locomotor hypotheses in extinct mammals.

## Author Contributions

Paul Medina‐González is the sole author of this manuscript. He was responsible for the conception, data collection, analysis, interpretation, figure preparation, manuscript writing, and final approval of the submitted version.

## Ethics Statement

Observations of walking movement patterns in animals were conducted under an approved animal care protocol issued by the Institutional Committee for the Care and Use of Laboratory Animals (CICUAL) of the Universidad Católica del Maule (Approval Certificate No. 10/2023), in accordance with national ethical regulations and within the framework of FONDECYT Project No. 11231111.

## Conflicts of Interest

The author declares no conflicts of interest.

## Supporting information

Supporting File 1.

Supporting File 2.

Supporting_File 3.

Supporting File 4.

Supporting File 5.

Supporting File 6.

Supporting File 7.

Supporting Figure S1.

## Data Availability

All data supporting the findings of this study are provided in the main text and in nine supporting files. The full data set and associated materials are archived in the Zenodo (DOI: 10.5281/zenodo.18164309). The repository includes species‐level records of posture classification, joint angular excursion (JAE), total angular excursion (TAE), angular utilization (AUI%), biological factor assignments, and derived statistical outputs (e.g., correlation and phylogenetic robustness results). Although the data set is already registered, file access is temporarily restricted during peer review. Access can be granted to reviewers on request and will become fully public upon publication.
