# Supplementary figures and images for: Joint Angular Excursions and Angular Range Utilization During Stance‐Phase Locomotion in Terrestrial Mammals: A Comparative Morphofunctional Data Set
Source: J Exp Zool A Ecol Integr Physiol. 2026 Feb 9;345(4):361–76. doi: 10.1002/jez.70069 (PMC13054777; doi:10.1002/jez.70069)

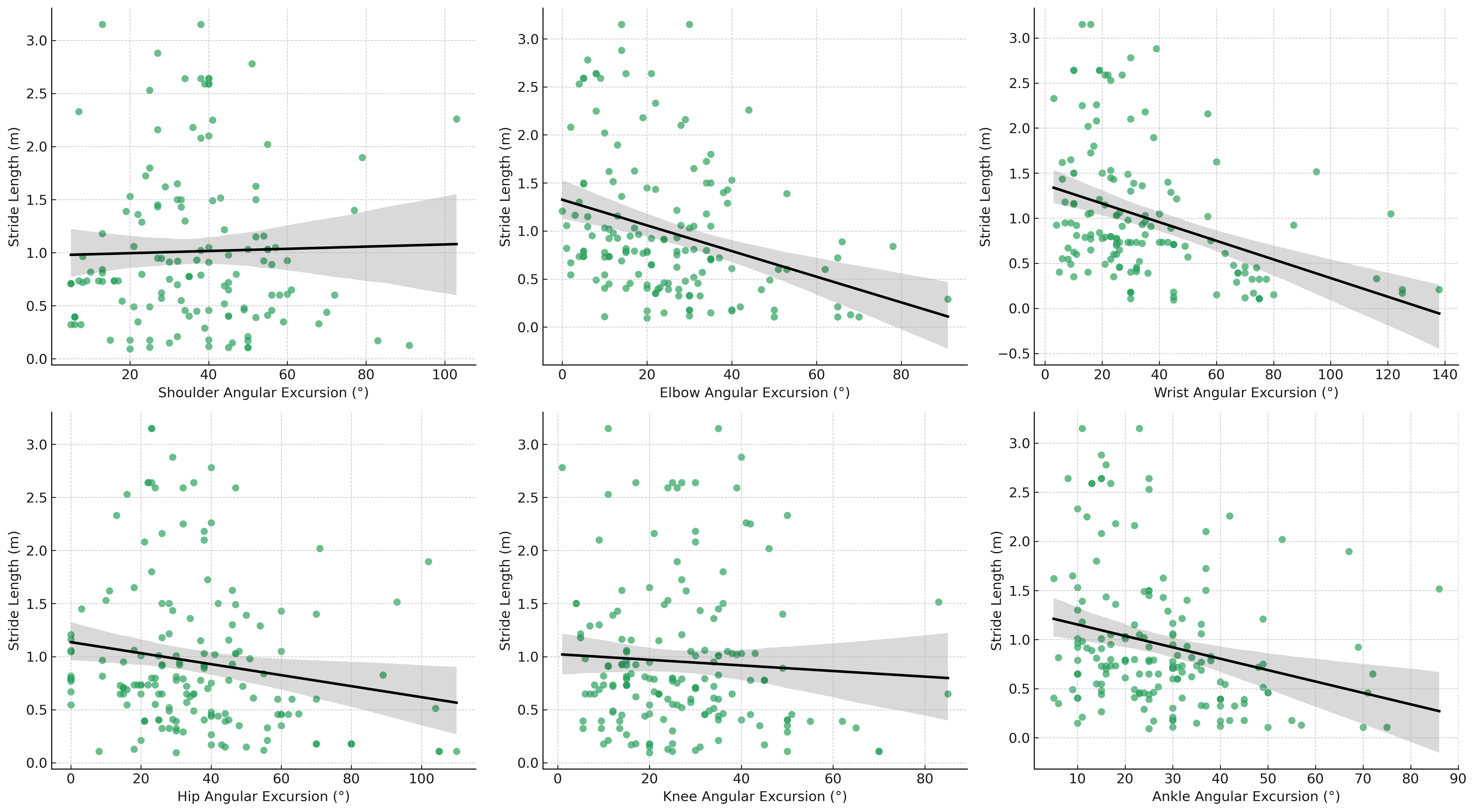

Supplement: Supplementary file 8 — Supporting Figure S1. [file JEZ-345-361-s006.tiff]
